# Evaluating the effectiveness of the 13-valent pneumococcal conjugate vaccine and clinical and demographic characteristics on pneumococcal carriage density in young children in Papua New Guinea, Lao PDR, and Mongolia

**DOI:** 10.1186/s12879-025-12328-w

**Published:** 2025-12-13

**Authors:** Claire von Mollendorf, Isatou Jagne, Elizabeth A. Ashley, Christopher C. Blyth, Jocelyn Chan, Rebecca L. Ford, Mayfong Mayxay, E. Kim Mulholland, Tuya Mungun, Dorj Narangerel, Odgerel Tundev, Monica L. Nation, Cattram D. Nguyen, Belinda D. Ortika, Casey L. Pell, Joycelyn Sapura, Keoudomphone Vilivong, Jana Lai, Yuhang Zhang, David A. B. Dance, William S. Pomat, Catherine Satzke, Fiona M. Russell

**Affiliations:** 1 Infection, Immunity and Global Health, Murdoch Children’s Research Institute, Parkville, VIC Australia; 2 Department of Paediatrics, The University of Melbourne, Melbourne, VIC Australia; 3 Lao-Oxford-Mahosot Hospital–Wellcome Trust Research Unit (LOMWRU), Mahosot Hospital, Vientiane, Lao People’s Democratic Republic; 4 Centre for Tropical Medicine and Global Health, Nuffield Department of Medicine, University of Oxford, Oxford, UK; 5 Wesfarmers Centre for Vaccines and Infectious Diseases, The Kids Research Institute Australia, Perth, Australia; 6 School of Medicine, The University of Western Australia, Perth Children’s Hospital, Perth, Australia; 7 Department of Infectious Diseases and PathWest Department of Microbiology, Perth Children’s Hospital, Perth, Australia; 8 Infection and Immunity Unit, Papua New Guinea Institute of Medical Research (PNG IMR), Goroka, Papua New Guinea; 9 Unit for Health Evidence and Policy (UHEP), Institute of Research and Education Development, University of Health Sciences, Ministry of Health, Vientiane, Lao People’s Democratic Republic; 10 Saw Swee Hock School of Public Health, National University of Singapore, Singapore, Singapore; 11 Faculty of Infectious and Tropical Diseases, London School of Hygiene & Tropical Medicine, London, UK; 12 National Center for Communicable Diseases (NCCD), Ulaanbaatar, Mongolia; 13 Ministry of Health, Ulaanbaatar, Mongolia; 14 National Centre for Epidemiology & Population Health, Australian National University, Canberra, Australia; 15 Department of Microbiology and Immunology, Peter Doherty Institute for Infection and Immunity, The University of Melbourne, Melbourne, Australia

**Keywords:** Pneumococcal nasopharyngeal density, Children, Pneumococcal conjugate vaccines, Carriage

## Abstract

**Background:**

High nasopharyngeal pneumococcal carriage density is associated with severe pneumonia; however, little is known about factors that affect pneumococcal carriage density including pneumococcal vaccination. We describe pneumococcal density by clinical and demographic factors, and effect of 13-valent pneumococcal conjugate vaccine (PCV13) on density in Papua New Guinea (PNG), Lao People’s Democratic Republic (Lao PDR) and Mongolia, 3–6 years following national PCV13 introduction.

**Methods:**

Three prospective pneumococcal carriage surveillance studies enrolled children aged 2–59 months with acute respiratory infections in Lao PDR (2013–2019), and pneumonia in PNG (2016–2019) and Mongolia (2015–2019). Demographic and clinical factors were collected on interview and from medical records. Nasopharyngeal swabs were tested for pneumococci using *lytA* real-time quantitative PCR and molecular serotyping using DNA microarray. In unvaccinated children median pneumococcal carriage density was compared across relevant clinical and demographic factors using Wilcoxon rank sum or Kruskal Wallis tests. Quantile regression models were used to determine the association between pneumococcal density, vaccination status and number of PCV doses.

**Results:**

A total of 1009 (PNG), 532 (Lao PDR) and 621 (Mongolia) pneumococcal carriers were included. Of carriers with serotyping results, PCV13 serotype (VT) carriage was 36.1% (356/985) in PNG, 40.8% (189/463) in Lao PDR and 50.7% (270/532) in Mongolia. The median pneumococcal VT density was 6.25 log_10_GE/ml (genome equivalents per milliliter) (interquartile range [IQR] 5.66, 6.79) in PNG, 5.74 log_10_GE/ml (IQR 4.99, 6.40) in Lao PDR and 5.64 log_10_GE/ml (IQR 5.11, 6.32) in Mongolia. In PNG, Lao PDR and Mongolia, 54.4%, 51.1% and 34.9% pneumococcal carriers were fully vaccinated, respectively. There was no difference in VT pneumococcal density by relevant clinical and demographic factors in unvaccinated children. In PNG, VT density was slightly lower (-0.36, 95% confidence interval [CI] -0.61, -0.12; *p* = 0.004) among vaccinated compared with unvaccinated children, in particular those who received three doses (-0.37 95% CI -0.63, -0.10; *p* = 0.007). No differences were observed in Lao PDR and Mongolia.

**Conclusions:**

We demonstrated variable results across our three sites. Indirect PCV13 effects may have resulted in limited observed reductions in VT density in unvaccinated children. In PNG, PCV13 vaccination was associated with a decline in VT density.

**Trial registration:**

Not applicable.

**Supplementary Information:**

The online version contains supplementary material available at 10.1186/s12879-025-12328-w.

## Background

*Streptococcus pneumoniae* (the pneumococcus) is a significant cause of morbidity and mortality worldwide, which mainly affects children, the elderly and immunocompromised individuals [1]. Pneumococcal nasopharyngeal carriage is common in young children [1]. Whilst generally asymptomatic, it is a prerequisite for pneumococcal disease [1, 2] and transmission [3].

High pneumococcal density in the nasopharynx is associated with an increased risk of invasive pneumococcal disease and transmission [4]. Carriage density varies between individuals and is influenced by host characteristics including age and underlying health conditions [5, 6]. Other factors such as viral co-infection and multiple serotype carriage may increase pneumococcal carriage density [8–9]. There is no consistent evidence on the impact of pneumococcal conjugate vaccines (PCVs) on pneumococcal nasopharyngeal density among children under five years old or other age groups. A recent systematic review on factors that affect pneumococcal density found considerable heterogeneity in study design and laboratory methods which made direct comparison between studies difficult [10]. The review included ten studies, four randomised trials, four cross sectional surveys, one case control study and one retrospective cohort study [10]. Three studies used semiquantitative culture methods to estimate pneumococcal density and the remainder used quantitative real-time PCR (qPCR) targeting the *lytA* gene. Of the nine studies that explored differences in pneumococcal density, three studies reported higher density, three studies reported lower density, and three studies reported no difference among vaccinated compared with unvaccinated children [10]. Data on the effect of PCV schedules or booster doses on carriage density is limited, with no difference observed in one clinical trial in Vietnam across these different groups [11, 12].

This study aimed to describe pneumococcal carriage density by demographic and clinical characteristics and determine the effect of number of doses and being fully vaccinated with 13-valent PCV (PCV13) on overall, PCV13 (VT) and non-PCV13 (NVT) serotype pneumococcal density in Papua New Guinea (PNG), Lao People’s Democratic Republic (Lao PDR) and Mongolia.

## Methods

To explore the effect of PCV13 on pneumococcal density among children under five years old hospitalised with acute respiratory infections (ARIs) or pneumonia, we used data from a multi-country prospective carriage surveillance study in PNG, Lao PDR and Mongolia [13]. PNG and Lao PDR are classified as lower-middle income countries and Mongolia as an upper-middle income country, with under-five mortality varying from ~ 40 per 1000 live births in PNG and Lao PDR to 13 per 1000 live births in Mongolia in 2022 [14].

PCV13 was introduced into the routine infant vaccination program (3 + 0 schedule) in PNG in October 2014 without a catch-up vaccination campaign, with widespread use in the study area by late 2015 [13]. WHO/UNICEF coverage for the third dose of PCV13 was estimated at 35% in 2019 and 29% in 2023 [15]. Lao PDR introduced PCV13 into the national childhood vaccination program in October 2013, using a 3 + 0 schedule. During the initial roll-out, a catch-up vaccination campaign of three doses targeted infants up to 12 months of age [13]. Estimated coverage for the third dose of PCV13 was 81% in 2019 and 2023 [15]. Mongolia introduced PCV13 in a phased manner by district from 2016 using a 2 + 1 schedule. In the first two years of introduction, catch-up of two doses was given for children up to 24 months of age [16]. Estimated national coverage for the third dose of PCV13 was 49% in 2019 and 96% in 2023 [15].

### Study population

Our secondary data analysis included children aged 2–59 months from hospital-based surveillance studies with ARIs in Lao PDR and pneumonia in PNG and Mongolia. Standardised participant recruitment and data collection methods were utilised in the three study sites, with local adaptations in eligibility criteria as the surveillance studies were nested within other pre-existing studies [13] (see Supplementary file for eligibility criteria).

Data were obtained from the Eastern Highlands Provincial Hospital in PNG from April 2016 to December 2019, Mahosot Hospital in Vientiane, Lao PDR from December 2013 to December 2019 and two district hospitals (Songinokhairkhan and Sukhbaatar District Hospitals) in Ulaanbaatar, Mongolia from November 2015 to March 2019. Clinical and demographic data, vaccination status and risk factors were collected from enrolled participants in all three sites [13]. Only pre-COVID-19 data were included to avoid potential confounding effects from non-pharmaceutical interventions used during the pandemic [17]. Only participants with detectable *S. pneumoniae* in the nasopharynx were included in the density analyses.

### Laboratory methods

Laboratory methods for the main studies have been previously described [18–20] and are outlined in the appendix. In brief, nasopharyngeal samples were collected using paediatric flocked swabs (Copan Diagnostics) according to the WHO recommendation [21]. Real-time qPCR targeting the *lytA* gene was used for pneumococcal detection. Carriage density (genome equivalents per milliliter, GE/ml) was determined using the average cycle threshold (Ct) value with reference to a standard curve prepared from genomic DNA of a reference isolate [18].

Molecular serotyping was conducted on the extracted DNA using Senti-SPv1.5 microarrays (BUGS Bioscience) [22]. PCV13 serotypes (VT serotypes) were defined as 1, 3, 4, 5, 6A, 6B, 7F, 9V, 14, 18C, 19A, 19F, and 23F. All other serotypes, including non-encapsulated pneumococci were considered non-PCV13 serotypes (NVT serotypes). Multiple serotype carriage was defined as a sample with more than one serotype, including non-encapsulated pneumococci, detected [18, 19]. To determine serotype-specific density (GE/ml), overall pneumococcal density (as determined by *lytA* qPCR) was multiplied by the percent relative abundance of each serotype (as determined by microarray). Density data were log transformed and reported as log_10_GE/ml.

### Vaccination status

Vaccination status was determined using evidence from written records, either parent-held immunisation records or health centre administrative records. Irrespective of age, children who received two or more doses of PCV13 were considered fully vaccinated and those who received one dose of PCV13 were considered partially vaccinated. Children who did not receive any dose of PCV13 vaccine constituted the unvaccinated group.

### Statistical analyses

Statistical analyses were performed using Stata version 18.5 (Stata Corp LP, College Station, TX). Data were analysed separately for each site. The distributions of pneumococcal density by various demographic characteristics among unvaccinated pneumococcal carriers in all three sites were visualised using violin plots. Unvaccinated children were used in this analysis to avoid potential confounding by PCV13 receipt. Median pneumococcal carriage density was compared across groups using Wilcoxon rank sum or Kruskal Wallis tests, as appropriate, to determine whether overall, VT and NVT density differed by age category (2, 3–5, 6–11, 12–23 and 24–59 months), number of serotypes (single or multiple serotypes), antibiotic use prior to admission (30 days in PNG, 7 days in Lao PDR, 48 h in Mongolia), household crowding (< 3 or ≥ 3 people per room in the dwelling), pneumonia severity (based on 2013 WHO definition [23]), type of cooking fuel used (wood/coal or electricity/gas), smoker in house, season (wet or dry in PNG/Lao PDR, cold or warm in Mongolia) and malnutrition status (malnourished [weight-for-age < -2 standard deviations of the WHO Child growth standards median] or well-nourished). Participant characteristics were stratified by vaccination status (fully vaccinated, partially vaccinated and unvaccinated) and summarised as counts and percentages.

Quantile regression models were used to determine the association between pneumococcal density, vaccination status and number of PCV doses. The multivariable models included covariates identified as potential confounders (age, crowding, season) using a directed acyclic graph (DAG) informed by relevant literature (Supplementary Fig. 1).

## Results

The characteristics of children included from all three sites by vaccination status are summarised in Table 1 with 1009, 532 and 621 pneumococcal carriers included in the analyses from PNG, Lao PDR and Mongolia, respectively. Unvaccinated children were noted to be slightly older than vaccinated children across all three sites (Table 1). Several differences were observed between the sites. In PNG the median age was 11 months (IQR 6–18), 15 months (IQR 8–25) in Lao PDR and 14 months (IQR 7–24) in Mongolia. Compared with children in PNG, children in Lao PDR and Mongolia used less wood or smoky fuel for cooking (96.8% vs. 39.6% and 69.8%), had lower rates of multiple serotype carriage (56.2% vs. 14.7% and 15.4%) and severe pneumonia (51.0% vs. 32.7% and 33.9%) and had lower median pneumococcal VT density. In Mongolia, a lower percentage of households had a sibling under five years of age (36.6% vs. 89.9% PNG and 99.6% Lao PDR) (Supplementary Table 1).

In all children across the three sites pneumococcal carriage was 91.5% (1009/1103) in PNG, 36.2% (532/1469) in Lao PDR, and 48.4% (621/1283) in Mongolia. Of those with known PCV status, the proportion of children who had received any vaccination were similar in all three sites between those who had detectable pneumococci on nasopharyngeal swab testing (carriers) and those that were deemed non-carriers. In PNG, 60.7% (549/904) of carriers and 56.2% (45/80) of non-carriers were vaccinated, in Lao PDR 53.4% (272/509) vs. 56.8% (507/893) and Mongolia 47.3% (286/605) vs. 52.9% (345/652), respectively.

Among all pneumococcal carriers, 985 (97.6%), 463 (87.0%), and 532 (85.7%) *S. pneumoniae* isolates were able to be serotyped from PNG, Lao PDR and Mongolia, respectively. Of those with serotyping results VT carriage was 36.1% (356/985) in PNG, 40.8% (189/463) in Lao PDR and 50.7% (270/532) in Mongolia. Median pneumococcal VT density was 6.25 log_10_GE/ml (IQR 5.66, 6.79) in PNG, 5.74 log_10_GE/ml (IQR 4.99, 6.40) in Lao PDR and 5.64 log_10_GE/ml (IQR 5.11, 6.32) in Mongolia (Supplementary Table 1).

**Table 1:** Characteristics of children aged 2–59 months with acute respiratory infection and Pneumococcal carriage by vaccination status in Papua new Guinea (April 2016 - December 2019), Lao people’s Democratic Republic (December 2013 - December 2019) and Mongolia (November 2015 - March 2019)

	Papua New Guinea (*N* = 1009)			Lao People’s Democratic Republic (*N* = 532)			Mongolia (*N* = 621)		
	Fully vaccinated^a^	Partially vaccinated ^a^	Unvaccinated ^a^	Fully vaccinated^a^	Partially vaccinated ^a^	Unvaccinated ^a^	Fully vaccinated^a^	Partially vaccinated ^a^	Unvaccinated ^a^
**Characteristics** **n (%)**	*n* = 549 (54.4)	*n* = 105 (10.4)	*n* = 355 (35.2)	*n* = 272 (51.1)	*n* = 23 (4.4)	*n* = 237 (44.5)	*n* = 217 (34.9)	*n* = 69 (11.1)	*n* = 319 (51.4.)
Age (months), median (IQR)	10 (6–15)	8 (5–15)	13 (7–32)	12 (8–20)	8 (3–19)	20 (10–32)	14 (9–22)	6 (3–11)	15 (8–28)
2–11, n (%)	322 (58.6)	66 (62.9)	162 (45.6)	123 (45.2)	13 (56.5)	63 (26.6)	79 (36.4)	52 (75.4)	124 (38.9)
12–23, n (%)	174 (31.7)	23 (21.9)	79 (22.3)	97 (35.7)	6 (26.1)	76 (32.1)	93 (42.9)	10 (14.5)	91 (28.5)
24–59, n (%)	53 (9.7)	16 (15.2)	114 (32.1)	52 (19.1)	4 (17.4)	98 (41.3)	45 (20.7)	7 (10.1)	104 (32.6)
Male sex, n (%)	304 (55.4)	60 (57.1)	211 (59.4)	149 (54.8)	11 (47.8)	125 (52.7)	119 (54.8)	45 (65.2)	171 (53.6)
**Household features**									
Living with ≤ 1 other child aged under five years, n (%)	495 (90.2)	99 (94.3)	313 (88.2)	*n* = 270	*n* = 23	*n* = 236	*n* = 212	*n* = 64	*n* = 311
				269 (99.6)	23 (100)	235 (99.6)	79 (37.3)	25 (39.1)	111 (35.7)
Crowding, n (%)^b^	*n* = 549	*n* = 103	*n* = 353	*n* = 271	*n* = 23	*n* = 236	*n* = 208	*n* = 65	*n* = 309
	180 (32.8)	47 (45.6)	125 (35.4)	57 (21.0)	9 (39.1)	79 (33.5)	69 (33.2)	26 (40.0)	94 (30.4)
Maternal education				*n* = 245	*n* = 20	*n* = 225	*n* = 213	*n* = 66	*n* = 314
Primary school or higher, n (%)^c^	-	-	-	231 (94.3)	19 (95.0)	201 (89.3)	213 (100.0)	66 (100.0)	312 (99.4)
Living below the poverty line, n (%)^d^	-	-	-	*n* = 272	*n* = 23	*n* = 237	*n* = 211	*n* = 62	*n* = 302
				72 (26.5)	1 (4.3)	65 (27.4)	50 (23.7)	16 (25.8)	64 (21.2)
Source of cooking fuel	*n* = 547	*n* = 104	*n* = 351	*n* = 231	*n* = 20	*n* = 148	*n* = 213	*n* = 67	*n* = 314
Wood or smoky fuel, n (%)	529 (96.7)	102 (98.1)	339 (96.6)	85 (36.8)	12 (60.0)	61 (41.2)	148 (69.5)	52 (77.6)	213 (67.8)
Season	237 (43.2)	48 (45.7)	144 (40.6)	*n* = 272	*n* = 23	*n* = 237	*n* = 217	*n* = 69	*n* = 321
Wet or cold, n (%)^e^				139 (51.1)	13 (56.5)	128 (54.0)	105 (48.4)	22 (31.9)	166 (52.0)
**Clinical features**	65 (11.8)	17 (16.2)	75 (21.1)	*n* = 267	*n* = 23	*n* = 232	*n* = 216	*n* = 69	*n* = 314
Malnutrition, n (%)^f^				48 (18.0)	4 (17.4)	46 (19.8)	10 (4.6)	2 (2.9)	23 (7.3)
	*n* = 544	*n* = 102	*n* = 353	*n* = 252	*n* = 21	*n* = 216	*n* = 214	*n* = 66	*n* = 303
Severe pneumonia, n (%)^g^	271 (49.8)	62 (60.8)	177 (50.1)	67 (26.6)	8 (38.1)	80 (37.0)	54 (25.2)	25 (37.9)	118 (38.9)
	*n* = 548	*n* = 105	*n* = 355	*n* = 267	*n* = 22	*n* = 227	*n* = 216	*n* = 69	*n* = 317
Prior antibiotic use, n (%)^h^	6 (1.1)	2 (1.9)	4 (1.1)	123 (46.1)	10 (45.4)	113 (49.8)	99 (45.8)	31 (44.9)	152 (47.9)
Multiple serotype carriage	*n* = 539	*n* = 101	*n* = 345	*n* = 241	*n* = 19	*n* = 203	*n* = 186	*n* = 55	*n* = 277
>=2 serotypes	288 (53.4)	56 (55.4)	210 (60.9)	34 (14.1)	5 (26.3)	29 (14.3)	27 (14.5)	8 (14.5)	44 (15.9)
**Pneumococcal density**	*n* = 549	*n* = 105	*n* = 355	*n* = 272	*n* = 23	*n* = 237	*n* = 217	*n* = 69	*n* = 319
**Overall carriage** Median log_10_GE/ml (IQR)	6.48 (5.84–7.03)	6.67 (6.08–7.04)	6.51 (6.01–7.05)	5.70 (5.02–6.32)	5.73 (5.09–6.35)	5.57 (4.97–6.25)	5.59 (5.08–6.43)	5.70 (5.08–6.13)	5.72 (5.17–6.33)
**VT carriage** Median log_10_GE/ml (IQR)	*n* = 149	*n* = 37	*n* = 170	*n* = 73	*n* = 7	*n* = 105	*n* = 67	*n* = 28	*n* = 169
	6.06 (5.44–6.69)	6.05 (5.71–6.69)	6.39 (5.95–6.92)	5.93 (5.01–6.33)	5.87 (4.44–6.35)	5.65 (4.99–6.45)	5.59 (5.09–6.42)	5.31 (4.74–6.12)	5.67 (5.22–6.27)
**NVT carriage** Median log_10_GE/ml (IQR)	*n* = 488	*n* = 86	*n* = 283	*n* = 175	*n* = 15	*n* = 113	*n* = 48	*n* = 5	*n* = 23
	6.40 (5.75–6.99)	6.64 (6.08–7.04)	6.39 (5.75–6.94)	5.65 (4.87–6.34)	5.78 (4.89–6.36)	5.61 (4.84–6.22)	6.01 (5.24–6.51)	5.94 (5.70–6.34)	5.32 (5.06–6.20)

a - Fully vaccinated: received ≥ 2 doses of PCV13, Partially vaccinated: received 1 dose of PCV13, Unvaccinated: no dose of PCV13 received irrespective of child’s age; b - Crowding defined as more than 3 people per sleeping room in the house; c - Higher than primary education only applies to Mongolia (3.3% completed primary, 50.7% completed secondary and 45.6% completed tertiary education); d - Below poverty line in Laos PDR less than US$1.25 (2013–2015) and US$1.90 (2016–2019) per person per day; in Mongolia household income ≤ 170,000₮; e - Wet season (PNG) refers to period from December to April; Wet season (Lao PDR) refers to period from May to October; Cold season (Mongolia) refers to period from November to March; f - Weight for age z score < 2SD below the mean; g - WHO 2013 severe pneumonia definition; h - Parent-reported antibiotic use 30 days before enrolment in PNG, 7 days before admission in Lao PDR and 48 h prior to admission in Mongolia

### Density by demographic factors in PCV unvaccinated children

Among unvaccinated pneumococcal carriers in PNG, Lao PDR and Mongolia, there were no obvious differences in overall pneumococcal density by prior antibiotic use, household crowding, pneumonia severity, type of cooking fuel used, smoker in the household, season or malnutrition status (Supplementary Figures S2A, S3A and S4A). In the Mongolia site only (Figure S4A), higher overall pneumococcal density (*p* = 0.008) was observed in those with multiple serotype carriage (6.10 [IQR 5.62–6.53 log_10_GE/ml]) versus single serotype carriage (5.70 [IQR 5.17–6.34 log_10_GE/ml]). Also in the Mongolia site, overall pneumococcal density was slightly higher in older children, with the highest density in the 6–11 month age group (*p* = 0.048): 2 months (5.11, IQR 4.55–5.35 log_10_GE/ml), 3–5 months (5.50, IQR 5.11–6.34 log_10_GE/ml), 6–11 months (5.80, IQR 5.45–6.39 log_10_GE/ml), 12–23 months (5.76, IQR 5.29–6.30 log_10_GE/ml) and 24–59 months (5.67, IQR 5.11–6.35 log_10_GE/ml). There were also no observed differences in VT and NVT density across any of the recorded factors in the three sites (Supplementary Figs. 2B/C, 3B/C and 4B/C).

### Density of individual serotypes

**Fig. 1:**
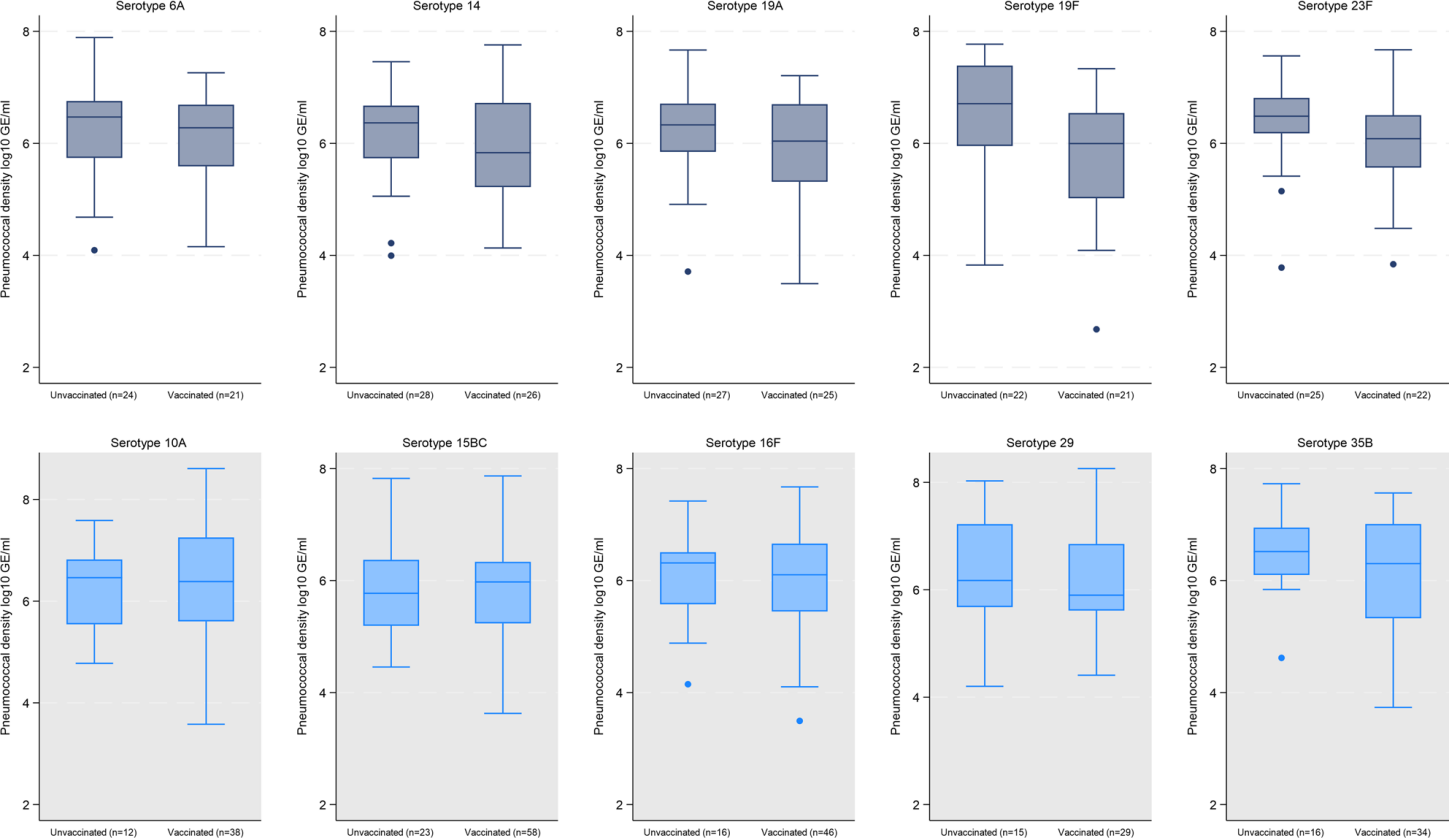
Serotype-specific density distribution of some of the common serotypes by PCV13 vaccination status in children with pneumonia and pneumococcal carriage in Papua New Guinea. PCV13 VTs = Gray, non-PCV13 VTs = Blue. Boxes depict the interquartile range (IQR) with a central line at the median, and whiskers extend 1.5 times the IQR past the quartiles. Values outside whiskers plotted as individual points

**Fig. 2:**
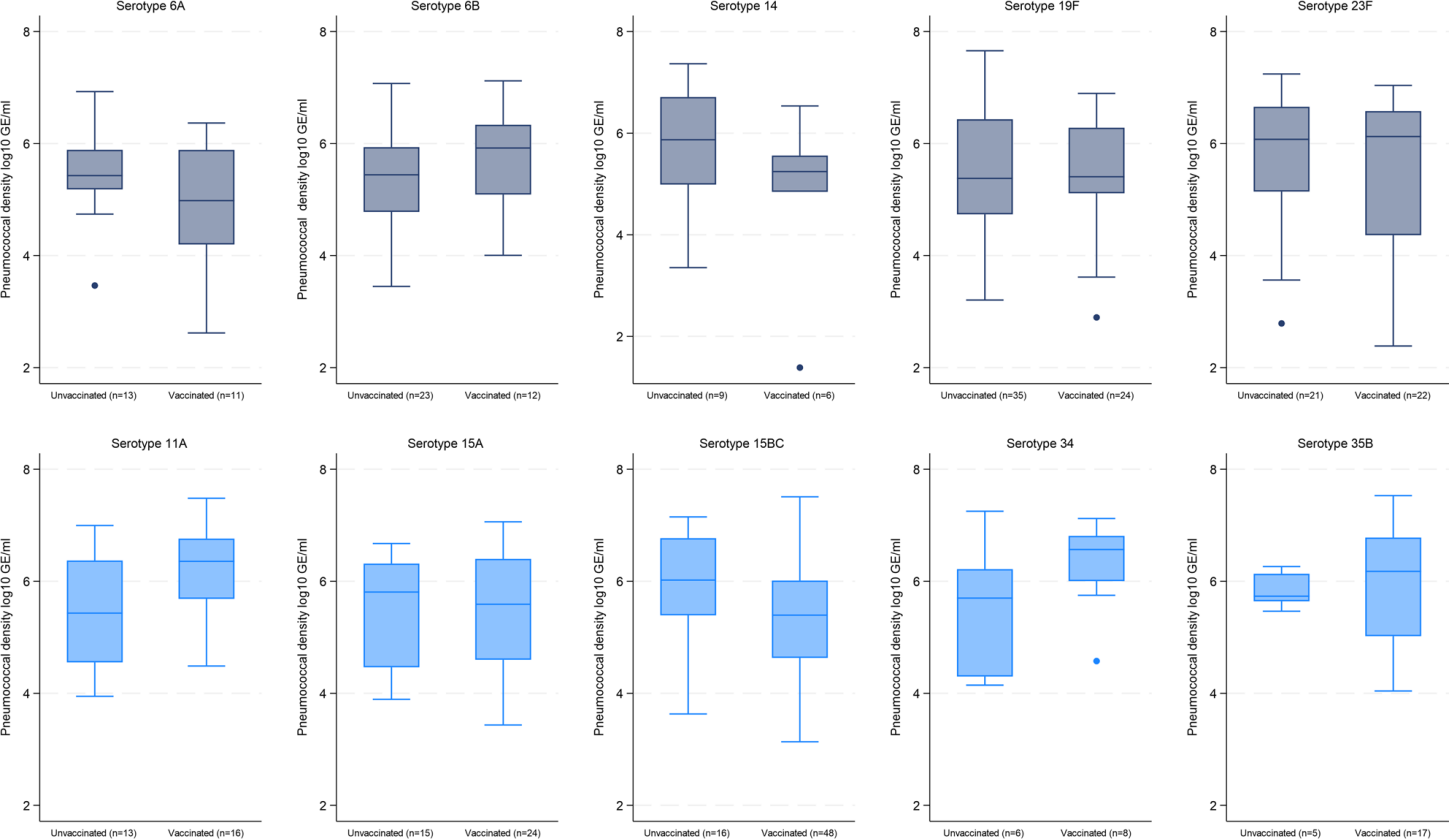
Serotype-specific density distribution of some of the common serotypes by PCV13 vaccination status in children with acute respiratory infection and pneumococcal carriage in Lao People’s Democratic Republic. PCV13 VTs = Gray, non-PCV13 VTs = Blue. Boxes depict the interquartile range (IQR) with a central line at the median, and whiskers extend 1.5 times the IQR past the quartiles. Values outside whiskers plotted as individual points

**Fig. 3:**
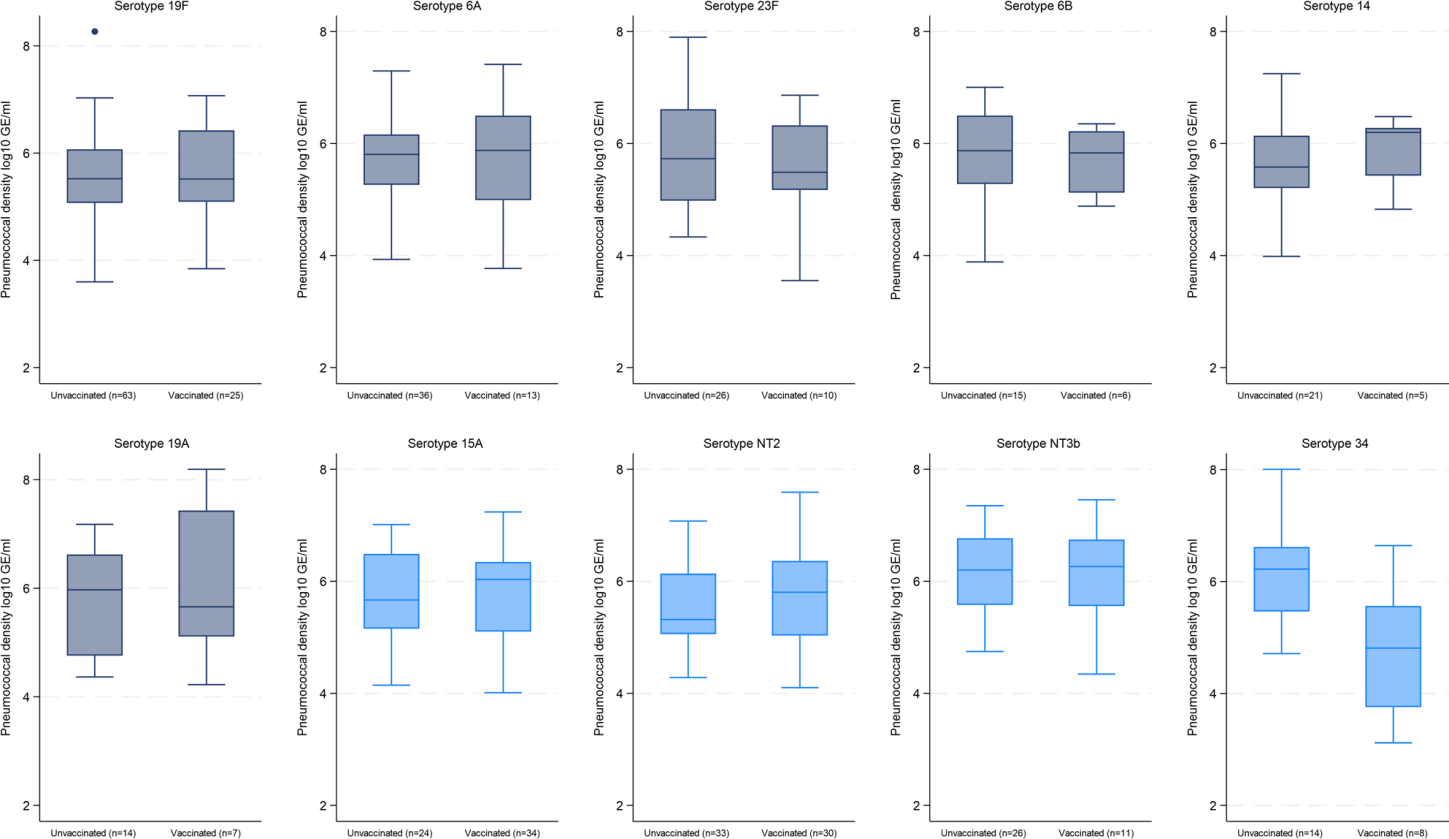
Serotype-specific density distribution of some of the common serotypes by PCV13 vaccination status in children with pneumonia and pneumococcal carriage in Mongolia. PCV13 VTs = Gray, non-PCV13 VTs = Blue. Boxes depict the interquartile range (IQR) with a central line at the median, and whiskers extend 1.5 times the IQR past the quartiles. Values outside whiskers plotted as individual points

A total of 68, 32, 37 unique individual serotypes were identified in PNG, Lao PDR and Mongolia, respectively. When we examined the top ten serotypes within each of the three sites, the serotype-specific pneumococcal density was generally similar between vaccinated and unvaccinated children for each serotype (Figs. 1, 2 and 3). In Lao PDR (Fig. 2), serotype 15B/C density was higher in unvaccinated children (*n* = 16; 6.02 [IQR 5.39–6.77 log_10_GE/ml]) compared with vaccinated children (*n* = 48; 5.39 [IQR 4.63–6.01 log_10_GE/ml], *p* = 0.02). In Mongolia (Fig. 3), serotype 34 density was higher in unvaccinated (*n* = 14; 6.22 [IQR 5.46–6.62 log_10_GE/ml]) compared with vaccinated children (*n* = 8; 4.81 [IQR 3.76–5.56 log_10_GE/ml], *p* = 0.01), although numbers were small.

For samples where pneumococci could be serotyped, multiple serotype carriage in PNG was 56.2% (554/985), 14.7% (68/463) in Lao PDR and 15.4% (82/532) in Mongolia. The overall pneumococcal density of individual serotypes for some of the most common serotypes was compared between children with multiple and single serotype carriage. Children with multiple serotype carriage generally exhibited similar overall pneumococcal densities compared with those where a single serotype only was detected, irrespective of PCV status (Supplementary Figure S5A, S5B and S5C). A few differences were observed in serotype-specific density, but numbers were small. Compared to single serotype carriers, in PNG multiple serotype carriage density was lower in unvaccinated children for serotype 15B/C (6.79 [IQR 6.37–7.52 log_10_GE/ml] vs. 5.71 [IQR 5.20–6.30 log_10_GE/ml], *p* = 0.03) and serotype 23 F (6.98 [IQR 6.82–7.48 log_10_GE/ml] vs. 6.31 [IQR 5.87–6.51 log_10_GE/ml], *p* = 0.002) and higher in unvaccinated children for serotype 35B (5.90 [IQR 5.23–6.21 log_10_GE/ml] vs. 6.72 [IQR 6.35–7.09 log_10_GE/ml], *p* = 0.03) (Supplementary Figure S5A). In Lao PDR, compared with children with single serotype carriage, multiple serotype carriage density was lower in unvaccinated (5.84 [IQR 5.27–6.56 log_10_GE/ml] vs. 4.74 [IQR 3.89–5.38 log_10_GE/ml], *p* = 0.004) and vaccinated (6.04 [IQR 5.20–6.45 log_10_GE/ml] vs. 5.17 [IQR 4.58–5.46 log_10_GE/ml], *p* = 0.04) children for serotype 19 F, and lower in vaccinated children for serotype 23 F (6.28 [IQR 6.00–6.62 log_10_GE/ml] vs. 3.95 [IQR 3.82–4.36 log_10_GE/ml], *p* = 0.03) (Supplementary Figure S5B). In Mongolia compared with children with single serotype carriage, multiple serotype carriage density was higher in unvaccinated children for NT2 (5.18 [IQR 4.86–5.59 log_10_GE/ml] vs. 5.92 [IQR 5.48–6.44 log_10_GE/ml], *p* = 0.02) and lower in vaccinated children for serotype 34 (5.15 [IQR 4.57–5.88 log_10_GE/ml] vs. 3.35 [IQR 3.12–3.58 log_10_GE/ml], *p* = 0.04) (Supplementary Figure S5C).

### Density and PCV13 status

The percentage of pneumococcal carriers that were fully vaccinated was 54.4% (549/1009), 51.1% (272/532) and 34.9% (217/621) for PNG, Lao PDR and Mongolia, respectively. In contrast 35.2% (355/1009), 44.5% (237/532) and 51.4% (319/621) were unvaccinated, respectively (Table 1).

There were no differences in overall pneumococcal density [-0.10 95% CI (-0.25, 0.04) *p* = 0.16)] and NVT density [-0.01 95% CI (-0.19, 0.17) *p* = 0.91] by vaccine status in PNG. In contrast, VT density was slightly lower [-0.36 95% CI (-0.61, -0.12); *p* = 0.004] among vaccinated children compared with unvaccinated children in PNG (Table 2). In Lao PDR and Mongolia, there were no differences in overall pneumococcal density [0.12 95% CI (-0.12, 0.36) and − 0.12 95% CI (-0.33, 0.08)], VT density [0.11 95% CI (-0.38, 0.60) and − 0.14 95% CI (-0.45, 0.16)] and NVT density [0.08 95% CI (-0.25, 0.42) and 0.30 95% CI (-0.36, 0.97)], respectively between PCV13 fully vaccinated and unvaccinated children (Table 2). Stratification by disease severity showed no difference in overall pneumococcal density by vaccine status in PNG, Laos or Mongolia (Supplementary Table 2).

In PNG (Table 3), overall [-0.15 95% CI (-0.29, -0.004); *p* = 0.04] and VT pneumococcal density [-0.37 95% CI (-0.63, -0.10); *p* = 0.007] were reduced for children receiving three doses of PCV13 compared with unvaccinated children. Point estimates were lower and confidence intervals were wide with no observable effect on overall or VT density for one and two doses. There was no difference by number of PCV13 doses for NVT pneumococcal density in PNG. In Lao PDR and Mongolia, there was no obvious difference by number of PCV13 doses for overall, VT or NVT pneumococcal density (Table 3).

**Table 2:** Quantile regression of Pneumococcal carriage density in PCV13^a^ vaccinated and unvaccinated children in three countries^b^

			Number of pneumococcal carriers	Median density (IQR)^c^	Adjusted coefficient (95% CI)^d^	*p* value
**Papua New Guinea (** ***n*** *** = 902)***						
Overall pneumococci^e^		Unvaccinated	353	6.51 (6.01–7.05)	Reference	
		PCV13-vaccinated	549	6.48 (5.84–7.03)	-0.10 (-0.25, 0.04)	0.16
Vaccine-type serotypes		Unvaccinated	169	6.39 (5.95–6.89)	Reference	
		PCV13-vaccinated	149	6.06 (5.44–6.69)	-0.36 (-0.61, -0.12)	0.004
Non-vaccine-type serotypes		Unvaccinated	282	6.39 (5.75–6.94)	Reference	
		PCV13-vaccinated	488	6.40 (5.75–6.99)	-0.01 (-0.19, 0.17)	0.91
**Lao PDR (** ***n*** ** = 507)**						
Overall pneumococci^e^		Unvaccinated	236	5.56 (4.97–6.26)	Reference	
		PCV13-vaccinated	271	5.70 (5.02–6.33)	0.12 (-0.12, 0.36)	0.33
Vaccine-type serotypes		Unvaccinated	104	5.66 (4.99–6.49)	Reference	
		PCV13-vaccinated	73	5.93 (5.01–6.33)	0.11 (-0.38, 0.60)	0.66
Non-vaccine-type serotypes		Unvaccinated	112	5.61 (4.88–6.22)	Reference	
		PCV13-vaccinated	174	5.65 (4.91–6.34)	0.08 (-0.25, 0.42)	0.61
**Mongolia (** ***n*** ** = 517)**						
Overall pneumococci^e^		Unvaccinated	309	5.70 (5.18–6.33)	Reference	
		PCV13-vaccinated	208	5.58 (5.08–6.42)	-0.12 (-0.34, 0.10)	0.27
Vaccine-type serotypes		Unvaccinated	165	5.67 (5.22–6.29)	Reference	
		PCV13-vaccinated	62	5.58 (5.09–6.35)	-0.14 (-0.45, 0.17)	0.38
Non-vaccine-type serotypes		Unvaccinated	23	5.32 (5.06–6.20)	Reference	
		PCV13-vaccinated	45	6.03 (5.25–6.47)	0.30 (-0.36, 0.97)	0.37

^a^PCV13=13-valent pneumococcal conjugate vaccine; ^b^Only children 2–59 months with known vaccination status were included. PCV13 vaccinated received ≥ 2 doses of PCV13 and unvaccinated received no PCV13 dose. Reported numbers are those included in adjusted analysis; ^c^Density reported in log_10_GE/ml; ^d^Coefficient is the difference in medians determined by quantile regression adjusted for age, household crowding, season; ^e^Overall pneumococci are not equal to the sum of vaccine-type and non- vaccine type serotypes. This is a result of multiple serotype carriage and/or exclusion of pneumococcal-positive samples for which a serotype was not determined

**Table 3:** Quantile regression of Pneumococcal carriage density in children who received 0–3 doses of PCV13^a^ in three countries^b^

		Number of pneumococcal carriers	Adjusted Coefficient (95% CI)^c^	*p* value
**Papua New Guinea (** ***n*** ** = 1005)**				
Overall pneumococci^d^	3 doses	392	-0.15 (-0.29, -0.004)	0.04
	2 doses	157	-0.05 (-0.24, 0.14)	0.60
	1 dose	103	0.07 (-0.15, 0.29)	0.52
	0 dose	353	Reference	
Vaccine-type serotypes	3 doses	100	-0.37 (-0.63, -0.10)	0.007
	2 doses	49	-0.29 (-0.63, 0.06)	0.10
	1 dose	37	-0.36 (-0.74, 0.01)	0.06
	0 dose	169	Reference	
Non-vaccine-type serotypes	3 doses	350	-0.01 (-0.20, 0.17)	0.89
	2 doses	138	-0.02 (-0.26, 0.23)	0.90
	1 dose	85	0.22 (-0.06, 0.50)	0.13
	0 dose	282	Reference	
**Lao People’s Democratic Republic (** ***n*** ** = 530)**				
Overall pneumococci^d^	3 doses	239	0.18 (-0.05, 0.42)	0.13
	2 doses	32	-0.17 (-0.65, 0.31)	0.48
	1 dose	23	0.13 (-0.42, 0.68)	0.64
	0 dose	236	Reference	
Vaccine-type serotypes	3 doses	61	0.24 (-0.26, 0.74)	0.35
	2 doses	12	-0.80 (-1.70, 0.11)	0.08
	1 dose	7	-0.02 (-1.16, 1.13)	0.98
	0 dose	104	Reference	
Non-vaccine-type serotypes	3 doses	154	0.10 (-0.25, 0.46)	0.57
	2 doses	20	-0.30 (-0.97, 0.38)	0.39
	1 dose	15	0.18 (-0.59, 0.95)	0.64
	0 dose	112	Reference	
**Mongolia (** ***n*** ** = 581)**				
Overall pneumococci^d^	3 doses	66	-0.15 (-0.46, 0.16)	0.35
	2 doses	142	-0.09 (-0.32, 0.15)	0.47
	1 dose	65	-0.05 (-0.37, 0.28)	0.77
	0 dose	308	Reference	
Vaccine-type serotypes	3 doses	14	0.07 (-0.55, 0.70)	0.82
	2 doses	48	-0.14 (-0.50, 0.22)	0.44
	1 dose	25	-0.40 (-0.89, 0.08)	0.11
	0 dose	165	Reference	
Non-vaccine-type serotypes	3 doses	24	0.12 (-0.65, 0.89)	0.76
	2 doses	21	0.60 (-0.27, 1.48)	0.17
	1 dose	5	0.53 (-0.78, 1.84)	0.42
	0 dose	23	Reference	

^a^PCV13=13-valent pneumococcal conjugate vaccine; ^b^Children 2–59 months; ^c^Coefficient is the difference in medians determined by quantile regression adjusted for age, household crowding, season. Reported numbers are those included in adjusted analysis; ^d^Overall pneumococci are not equal to the sum of VT and NVT serotypes. This is a result of multiple serotype carriage and/or exclusion of pneumococcal-positive samples for which serotype was not determined

## Discussion

This multi-country study, which used similar recruitment methods and the same analytical and laboratory methods across sites, identified limited differences in overall, VT or NVT pneumococcal carriage density distribution in unvaccinated children. In PNG, a small reduction in VT pneumococcal density was found in vaccinated children, in particular those who received three doses of PCV13. No detectable impact of PCV13 on pneumococcal VT density was observed when comparing vaccinated to unvaccinated children in Lao PDR or Mongolia, possibly due to limited case numbers but likely also due to considerable indirect effects on unvaccinated children [24, 25].

Previous studies conducted across our three country sites have shown some differences in the impact of PCV13 on pneumococcal density. All these studies utilised the same laboratory methods as our study. A community cross-sectional survey conducted in Lao PDR within two years of the introduction of PCV13, reported higher VT and NVT pneumococcal density among vaccinees compared with unvaccinated healthy children. As both VT and NVT density increased these changes may not have been due to the vaccine [19]. A previous study conducted in PNG, involving a cohort of infants who received three doses of PCV13, indicated no discernible effect of PCV13 on VT density [20]. The difference in age group, study design, study years and PCV coverage likely account for the difference in results compared with our study. In Mongolia, previous studies have demonstrated an increase in all and VT pneumococcal carriage density both in children with pneumonia and healthy children following the introduction of PCV13^18,26^. The observed variations in findings may be due to the differences in populations (community versus hospital populations), age groups or time since PCV13 introduction.

A study from Nepal comparing pre- and post-PCV10 pneumococcal carriage data demonstrated a year-on-year decline in carriage density following PCV10 introduction [27]. No dosage effect on density was observed in Nepal, similar to our results from Lao PDR and Mongolia. The authors commented that the decline in density was unrelated to the number of PCV10 doses, suggesting the ongoing impact of the PCV10 program (i.e. indirect effects) rather than direct protective effects [27]. The importance of density on the effect of PCVs has been demonstrated in human challenge studies. A randomised trial investigating PCV13 efficacy in an adult human pneumococcal challenge model demonstrated that serotype 6B pneumococcal colonisation acquisition post-inoculation was reduced in the vaccinated versus the control group. In addition to the colonisation acquisition rate, a reduction was observed in the colonisation density in the vaccinated group [28].

Our study was undertaken in the context of PCV13 being introduced for three years in Mongolia, five years in PNG and six years in Lao PDR. In all three countries considerable indirect effects of PCV13 vaccination on pneumococcal carriage have been demonstrated [24–26, 29]. Findings in Lao PDR indicated that as PCV13 coverage increased from zero to 60%, the prevalence of PCV13 VT carriage decreased by 36%^24^. Similarly in Mongolia it was estimated that as coverage approached 100%, VT carriage would reduce by 55% through indirect effects alone [25]. In a community carriage survey in Mongolia, in infants too young to be vaccinated, VT pneumococcal carriage was reduced by 67% (compared to pre-vaccine prevalence) six years post-PCV13 introduction [26]. In PNG, despite much lower overall vaccine coverage rates (12–29%), indirect effects were also evident [29]. These indirect effects likely reduced differences between vaccinated and unvaccinated children in these settings.

Studies have shown reductions in multiple-serotype carriage following PCV introduction [26, 30]. PCV effects on density related to multiple serotype carriage is less well defined. No difference in overall pneumococcal density between children with single versus multiple serotype carriage was observed in PNG and Lao PDR, which aligns with findings from a study in Peru [31]. In Mongolia, similar to a study from Indonesia, an association between multiple serotype carriage and higher density was found [32]. The frequency of multiple serotype carriage varies between different pneumococcal serotypes and is therefore influenced by PCV status and the relative frequency of circulating serotypes [31]. This may explain some of the differences in multiple serotype carriage observed between sites.

Our analysis, which was restricted to unvaccinated sick children with ARI or pneumonia, found that VT density did not vary by age in any of the three countries. In contrast, a series of community carriage surveys in The Gambia reported a decline in all serotype density with increasing age; however, all children below 30 months of age had received PCV7, suggesting that some vaccine related effects may have contributed to these findings [33]. Pre-PCV introduction studies in Indonesia and Peru, found contrasting trends in pneumococcal carriage density by age [31, 32]. In Peru, in children < 3 years, densities increased with age to 6 months and then stabilised [31], whereas in Indonesia, carriage density in healthy children decreased slightly with age up to 12 months [32]. These differences highlight the dynamic changes seen in carriage density across different settings with variable sociodemographic factors and variations in cohorts (healthy versus sick). Other factors related to age, such as reduced carriage rates and increased immunity, may also influence density results.

We identified no difference in pneumococcal density between children who received or did not receive antibiotics prior to admission across our three sites. This lack of difference was observed in previous community carriage surveys conducted in Lao PDR [34], but differed from other studies which found lower carriage density among pneumonia cases pretreated with antibiotics [32, 35]. Variations in study designs, antibiotic exposure definitions, and/or sample collection/handling could all contribute to these different findings. In addition, ascertainment of prior antibiotic use was by parent recall, and this is likely to be prone to recall bias.

The greatest number of serotypes (diversity) was observed in PNG compared with the other two sites. PCV introduction drives serotype replacement, leading to an initial increase in diversity followed by potential shifts to non-vaccine types. Host and environmental factors as well as prolonged carriage durations have been associated with increased diversity. PNG had the highest proportion of risk factors, carriage prevalence and proportion of NVT serotypes of the three countries [20].

Our findings differed from studies, such as the PERCH study, in that we found no difference in median pneumococcal density between severe pneumonia cases and non-severe ARIs [35]. A similar case definition was used in the Laos study site while the PNG and Mongolia study sites used the WHO severe pneumonia case definition compared with non-severe cases. A study in Lao PDR using the same data analysed here, found a positive association between density and severe pneumonia, but only on adjusted analysis [36]. No association was observed on unadjusted analysis, which is similar to our findings. A previous study in Lao PDR showed that use of charcoal or wood for cooking and having a smoker in the home did not influence pneumococcal carriage density [34]. This is in line with findings from our three sites wherein we found no obvious difference in pneumococcal carriage density between children from households using different types of cooking fuels.

Our study had several strengths. We included three different countries to more comprehensively address the research question. We used consistent sample collection and laboratory methods across the three countries, reducing chance of result variation caused by introduction of different methodologies. We also utilised sensitive molecular methods to quantify pneumococcal density and identify multiple serotype carriage, including detection of serotypes in low abundance. These data are usually scarce in LMICs. We excluded data from the COVID pandemic period as previous research demonstrated a reduction in overall pneumococcal carriage density following non-pharmaceutical interventions despite stable carriage prevalence [17]. Our study also had some limitations. We could not account for all key confounders as they were not available for all three sites. This included viral co-detection which may influence pneumococcal density, and as study participants all had ARI or pneumonia at time of sampling, co-detection is likely to be substantial. For our multiple serotype carriage analysis some of the compared serotypes would have been minor serotypes which may have had different density to major serotypes. As nasopharyngeal swabs may be distressing to children, inadequate swabs may impact carriage and density measures. We ensured that staff were fully trained to reduce distress and to take adequate swabs. In addition, refusal rates were low in all sites. There were some differences between our three sites. PNG had higher carriage and density, lower coverage and reported fewer antibiotics than Lao PDR and Mongolia. In addition, the antibiotic use time period differed between the three sites. In Lao PDR and Mongolia, there was a lower rate of overall pneumococcal carriage which reduced case numbers included in this analysis. This potentially reduced our ability to detect small differences in density by vaccination status. There were substantial differences in density point estimates likely reflecting small numbers in some subgroups. As all children included in this analysis had ARI or pneumonia, the findings may not be generalisable to healthy paediatric populations. One study from Peru demonstrated a variation in pneumococcal density before, during and following an ARI episode [37]. In addition, characteristics and responses of vaccinated children who carry vaccine serotypes may differ from other children who carry other serotypes or who are not carriers. Lastly, as vaccine coverage increases the confounding effects of indirect vaccine impact reduces the differences in density between vaccinated and unvaccinated children.

## Conclusions

Our study demonstrated that among children under five years with pneumonia in PNG, vaccination with PCV13 reduced VT pneumococcal density. The magnitude of this reduction, however, was small and the clinical significance is therefore unclear. We demonstrated different results across our three sites despite using consistent sample collection and laboratory methods to enumerate density. These differences may have been due to differences in demographics, carriage rates, inclusion criteria such as ARI or pneumonia definition, vaccine coverage rates, time since vaccine introduction and indirect effects. This variation in results highlights the need to evaluate complex questions across different settings to verify findings.

## Supplementary Information

Below is the link to the electronic supplementary material.

Supplementary Material 1

## Data Availability

Data is provided within the manuscript or supplementary information files.

## Abbreviations

ARIs: Acute respiratory infections
95% CI: 95% confidence interval
COVID-19: Coronavirus disease 2019
Ct: Cycle threshold
DAG: Directed acyclic graph
GE/ml: Genome equivalents per milliliter
IQR: Interquartile range
Lao PDR: Lao People’s Democratic Republic
NVT serotypes: Non-PCV13 serotypes
PCV: Pneumococcal conjugate vaccine
PCV13: 13-valent pneumococcal conjugate vaccine
PNG: Papua New Guinea
qPCR: Quantitative real-time PCR
UNICEF: United Nations Children’s Fund
VT serotypes: PCV13 serotypes
WHO: World Health Organization
