# Supplementary material for: Evaluating the effectiveness of the 13-valent pneumococcal conjugate vaccine and clinical and demographic characteristics on pneumococcal carriage density in young children in Papua New Guinea, Lao PDR, and Mongolia
Source: BMC Infect Dis. 2025 Dec 13;26:97. doi: 10.1186/s12879-025-12328-w (PMC12821165; doi:10.1186/s12879-025-12328-w)
Supplement: Supplementary file 1 — Supplementary Material 1 [file 12879_2025_12328_MOESM1_ESM.docx]

**Supplementary material**

**Supplementary methods**

*Study procedures*

Consistent participant recruitment and data collection methods were utilised in the three study sites, with local adaptations in eligibility criteria as the surveillance studies were nested within other pre-existing studies^1^. The inclusion criteria for the main study in PNG included children with moderate pneumonia i.e. cough, tachypnoea (≥50 breaths per minute) and lower chest wall indrawing, with children living ≥1 hour outside town or hospitalised within 14 days of prior admission excluded. In Lao PDR, any child admitted to hospital with fever and either cough or dyspnoea or rhinitis or abnormal chest auscultation were eligible for inclusion. In Mongolia, children with pneumonia i.e. cough, or dyspnoea and tachypnoea (≥50 breaths per minute) or hypoxia or chest wall indrawing were included. Children admitted with pneumonia in the previous 14 days were excluded. Clinical and demographic data, vaccination status and risk factors were collected from enrolled participants in all three sites^1^.

*Laboratory methods*

Laboratory methods for the main studies have been previously described^2-4^. In brief nasopharyngeal samples were collected using paediatric flocked swabs (Copan Diagnostics) according to the WHO recommendation^5^. In brief, the swabs were placed into 1 ml of skim milk tryptone glucose glycerol (STGG) medium, transported to in-country laboratories where they were vortexed, dispensed into aliquots, and stored at ultra-low temperatures. The samples were batched and shipped on dry ice to the Murdoch Children’s Research Institute (Parkville, Australia) for laboratory testing. Real-time quantitative PCR (qPCR) targeting the *lytA* gene was used for pneumococcal detection. Carriage density (genome equivalents per milliliter, GE/ml) was determined using the average cycle threshold (Ct) in reference to a standard curve prepared from genomic DNA of a reference *Streptococcus pneumoniae* isolate^2^.

Following culture amplification, molecular serotyping was conducted on the extracted DNA using Senti-SPv1.5 microarrays (BUGS Bioscience). Analysis of the microarray data was accomplished using custom web-based software employing a Bayesian-based model^6^. *LytA* qPCR positive (Ct value <35) samples that were unable to be serotyped (due to negative culture or low DNA yield from culture), were considered pneumococcal positive, serotype unknown. PCV13 serotypes (VT serotypes) were defined as 1, 3, 4, 5, 6A, 6B, 7F, 9V, 14, 18C, 19A, 19F, and 23F. All other serotypes, including non-encapsulated pneumococci were considered non-PCV13 serotypes (NVT serotypes). Different lineages of non-encapsulated pneumococci include NT2, NT3b and NT4b^7^. Serotypes 15B and

15C were reported as 15B/C and 11F-like serotypes as 11A^7,8^. A swab containing both VT and NVT serotype(s) was considered positive for both. Multiple serotype carriage was defined as a sample where more than one serotype including non-encapsulated pneumococci was detected^2,3^.

To determine serotype-specific density (GE/ml), overall pneumococcal density (as determined by *lytA* qPCR) was multiplied by the percent relative abundance of each serotype (as determined by microarray). Density data were log transformed and reported as log_10_GE/ml.

**Supplementary figures and tables**


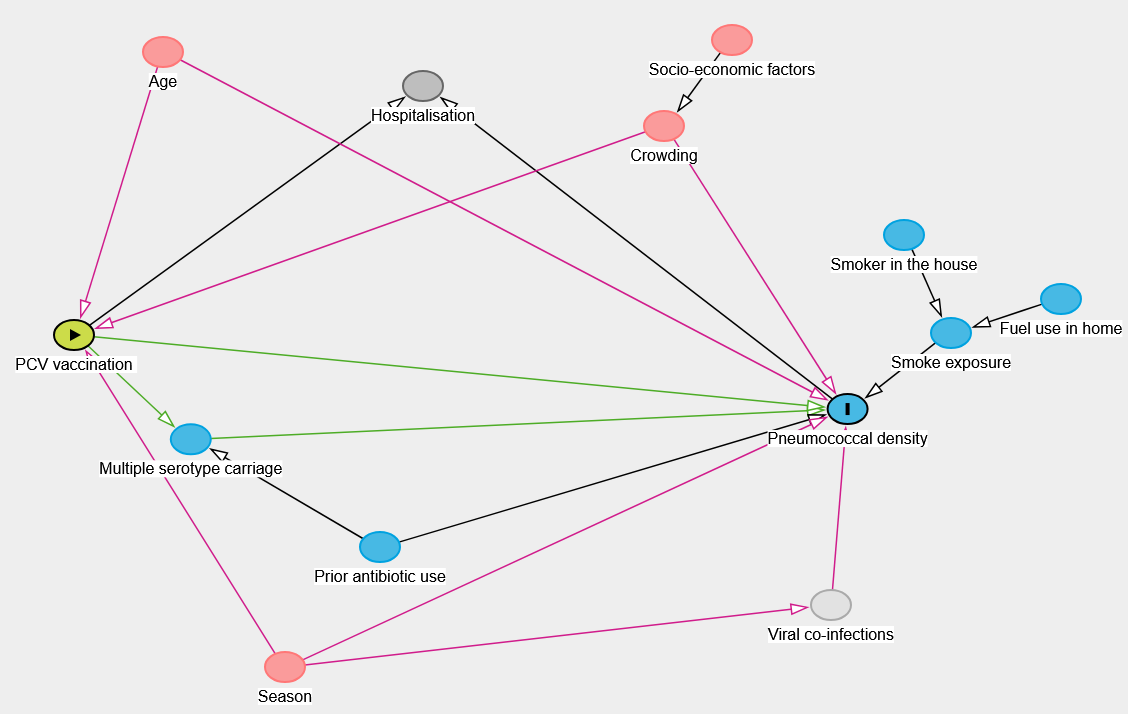


**Supplementary Figure S1:** Directed acyclic graph. Causal pathways identified in green. Biasing pathways identified as pink arrows. Variables in blue represent ancestors of the outcome density. Variables in pink represent ancestors of both the exposure and the outcome. Light grey variable represents unmeasured/unobserved variable. Dark grey represents a collider variable.

Variables in the DAG: PCV vaccination, pneumococcal colonisation density, age, multiple serotype carriage, prior antibiotic use, season, viral co-colonisation, household crowding with socioeconomic factors, and smoke exposure with smoker and fuel use in home, and hospitalisation.

**Supplementary Table 1: Characteristics of children aged 2-59 months with respiratory infections and pneumococcal carriage in three countries^a^**

|  | **Papua New Guinea (N=1009)** | **Lao PDR**  **(N=532)** | **Mongolia**  **(N=621)** |
| --- | --- | --- | --- |
| **Characteristics** |  |  |  |
| Age, in months, median (IQR) | 11 (6 – 18) | 15 (8 – 25) | 14 (7 – 24) |
| 2 - 11, n (%) | 550 (54.5) | 199 (37.4) | 258 (41.5) |
| 12 - 23, n (%) | 276 (27.4) | 179 (33.6) | 200 (32.2) |
| 24 - 59, n (%) | 183 (18.1) | 154 (29.0) | 163 (26.3) |
| Male sex, n (%) | 575 (57.0) | 285 (53.6) | 343 (55.2) |
| **Household features** |  |  |  |
| Living with ≥1 other child aged under five years, n (%) | 907 (89.9) | 527/529 (99.6) | 220/601 (36.6) |
| Crowding, n (%)^b^ | 352/1005 (35.0) | 145/530 (27.4) | 193/597 (32.3) |
| Maternal education: Completed primary school or higher, n (%)^c^ | Not collected | 451/495 (91.1) | 607/609 (99.7) |
| Living below the poverty line, n (%)^d^ | Not collected | 138 (25.9) | 133/590 (22.5) |
| Source of cooking fuel: Wood only or smoky fuel (coal/wood), n (%) | 970/1002 (96.8) | 158/399 (39.6) | 426/610 (69.8) |
| Season of sample collection: Wet or cold, n (%)^e^ | 429 (42.5) | 280 (52.6) | 296 (47.7) |
| **Clinical features** |  |  |  |
| Malnutrition, n (%)^f^ | 157 (15.6) | 98/522 (18.8) | 35/614 (5.7) |
| Severe pneumonia, n (%)^g^ | 510/999 (51.0) | 164/501 (32.7) | 203/599 (33.9) |
| Prior antibiotic use, n (%)^h^ | 12/1008 (1.2) | 246/518 (47.5) | 290/618 (46.9) |
| Multiple serotype carriage: ≥2 serotypes | 554/985 (56.2) | 68/463 (14.7) | 82/532 (15.4) |
| **Pneumococcal density** | n=1009 | n=532 | n=621 |
| **Overall carriage -** Median log_10_GE/ml (IQR) | 6.52 (5.95 - 7.04) | 5.65 (5.00 - 6.29) | 5.67 (5.13 - 6.35) |
|  | n= 356 | n=185 | n=270 |
| **VT carriage -** Median log_10_GE/ml (IQR) | 6.25 (5.66 - 6.79) | 5.74 (4.99 - 6.40) | 5.64 (5.11 - 6.32) |
|  | n=857 | n=303 | n=80 |
| **NVT carriage -** Median log_10_GE/ml (IQR) | 6.42 (5.77 - 6.98) | 5.64 (4.87 - 6.26) | 5.79 (5.12 - 6.45) |

^a^ Time period: Papua New Guinea (April 2016 - December 2019), Lao People’s Democratic Republic (December 2013 - December 2019) and Mongolia (November 2015 - March 2019); ^b^ Crowding defined as more than 3 people per sleeping room in the house; ^c^ Not collected for PNG. Higher than primary education only applies to Mongolia (3.3% completed primary, 50.7% completed secondary and 45.6% completed tertiary education); ^d^ Below poverty line in Lao PDR less than US$1.25 (2013-2015) and US$1.90 (2016-2019) per person per day; in Mongolia household income ≤170,000₮; ^e^ Wet season (PNG) refers to period from December to April, Wet season (Lao PDR) refers to period from May to October, Cold season (Mongolia) refers to period from November to March; ^f^ Weight for age z score < -2SD below the mean; ^g^ WHO 2013 severe pneumonia definition; ^h^ Parent report of antibiotic use 30 days before enrolment in PNG, 7 days before admission in Lao PDR and 48 hours prior to admission in Mongolia

**Supplementary Table 2: Quantile regression of pneumococcal carriage density by disease severity in PCV13^a^ vaccinated and unvaccinated children in three countries^b^**

|  | **Severity of disease^c^** | **Vaccination status** | **Number of pneumococcal carriers** | **Median density (IQR)^d^** | **Adjusted coefficient (95% CI)^e^** | **p value** |
| --- | --- | --- | --- | --- | --- | --- |
| **Papua New Guinea (n=895)** | | | | | | |
| **Overall pneumococci** | Not severe | Unvaccinated | 175 | 6.45 (6.11-6.97) | Reference |  |
|  |  | PCV13-vaccinated | 273 | 6.48 (5.91-7.12) | -0.07 (-0.26, 0.13) | 0.48 |
|  | Severe | Unvaccinated | 176 | 6.58 (5.93-7.11) |  |  |
|  |  | PCV13-vaccinated | 271 | 6.44 (5.80-6.99) | -0.13 (-0.36, 0.10) | 0.27 |
| **Lao PDR (n=478)** | | | | | | |
| **Overall pneumococci** | Not severe | Unvaccinated | 135 | 5.61 (4.99-6.32) | Reference |  |
|  |  | PCV13-vaccinated | 188 | 5.66 (5.02-6.31) | 0.01 (-0.29, 0.30) | 0.97 |
|  | Severe | Unvaccinated | 84 | 5.59 (4.96-6.26) | Reference |  |
|  |  | PCV13-vaccinated | 71 | 5.97 (5.18-6.35) | 0.39 (-0.08, 0.86) | 0.11 |
| **Mongolia (n=554)** | | | | | | |
| **Overall pneumococci** | Not severe | Unvaccinated | 179 | 5.67 (5.13-6.33) | Reference |  |
|  |  | PCV13-vaccinated | 208 | 5.66 (5.11-6.43) | -0.04 (-0.29, 0.22) | 0.77 |
|  | Severe | Unvaccinated | 115 | 5.75 (5.23-6.39) | Reference |  |
|  |  | PCV13-vaccinated | 52 | 5.46 (5.01-6.35) | -0.34 (-0.75, 0.08) | 0.11 |

^a^PCV13=13-valent pneumococcal conjugate vaccine; ^b^Only children 2-59 months with known vaccination status were included. PCV13 vaccinated received ≥2 doses of PCV13 and unvaccinated received no PCV13 dose. Reported numbers are those included in adjusted analysis; ^c^WHO 2013 severe pneumonia definition; ^d^Density reported in log10GE/ml; ^e^Coefficient is the difference in medians determined by quantile regression adjusted for age, household crowding, season

**

**Supplementary Figure S2A:** Distribution of overall pneumococcal carriage density (log_10_ GE/mL) by A) age in months (n=355), B) number of serotypes (n=345), C) prior antibiotic use (n=355), D) household crowding (n=353), E) pneumonia severity (n=353), F) type of cooking fuel used (n=351), G) smoker in house (n=353), H) season (n=355), and I) malnutrition status (n=355) among unvaccinated pneumococcal carriers in Papua New Guinea children aged 2-59 months with moderate pneumonia. The white dot represents the median, the black bar depicts the interquartile range (IQR) and whiskers extend 1.5 times the IQR past the quartiles.

**Supplementary Figure S2B:** Distribution of pneumococcal PCV13-type carriage density (log_10_ GE/mL) by A) age in months (n=170), B) number of serotypes (n=170), C) prior antibiotic use (n=170), D) household crowding (n=169), E) pneumonia severity (n=169), F) type of cooking fuel used (n=169), G) smoker in house (n=169), H) season (n=170), and I) malnutrition status (n=170) among unvaccinated pneumococcal carriers in Papua New Guinea children aged 2-59 months with moderate pneumonia.

The white dot represents the median, the black bar depicts the interquartile range (IQR) and whiskers extend 1.5 times the IQR past the quartiles.

**Supplementary Figure S2C:** Distribution of non-PCV13 type pneumococcal carriage density (log_10_ GE/mL) by A) age in months (n=283), B) number of serotypes (n=283), C) prior antibiotic use (n=283), D) household crowding (n=282), E) pneumonia severity (n=282), F) type of cooking fuel used (n=279), G) smoker in house (n=282), H) season (n=283), and I) malnutrition status (n=283) among unvaccinated pneumococcal carriers in Papua New Guinea children aged 12-59 months with moderate pneumonia. The white dot represents the median, the black bar depicts the interquartile range (IQR) and whiskers extend 1.5 times the IQR past the quartiles.

**Supplementary Figure S3A:** Distribution of overall pneumococcal carriage density (log_10_ GE/mL) by A) age in months (n=164), B) number of serotypes (n=132), C) prior antibiotic use (n=153), D) household crowding (n=159), E) pneumonia severity (n=142) F) type of cooking fuel used (n=82) G) smoker in house (n=159), H) season (n=159) and I) malnutrition status (n=156) among unvaccinated children aged 2-59 months with pneumococcal carriage admitted with acute respiratory infections in Lao People’s Democratic Republic. The white dot represents the median, the black bar depicts the interquartile range (IQR) and whiskers extend 1.5 times the IQR past the quartiles.

**

**Supplementary Figure S3B:** Distribution of pneumococcal PCV13-type carriage density (log_10_ GE/mL) by A) age in months (n=82), B) number of serotypes (n=79), C) prior antibiotic use (n=76), D) household crowding (n=79), E) pneumonia severity (n=75), F) type of cooking fuel used (n=37), G) smoker in house (n=79), H) season (n=79) and I) malnutrition status (n=79) among unvaccinated children aged 2-59 months with pneumococcal carriage admitted with acute respiratory infections in Lao People’s Democratic Republic. The white dot represents the median, the black bar depicts the interquartile range (IQR) and whiskers extend 1.5 times the IQR past the quartiles.

**Supplementary Figure S3C:** Distribution of non-PCV13 type pneumococcal carriage density (log_10_ GE/mL) by A) age in months (n=66), B) number of serotypes (n=63), C) prior antibiotic use (n=62), D) household crowding (n=63), E) pneumonia severity (n=53), F) type of cooking fuel used (n=33), G) smoker in house (n=63), H) season (n=63) and I) malnutrition status (n=60), among unvaccinated children aged 2-59 months with pneumococcal carriage admitted with acute respiratory infections in Lao People’s Democratic Republic. The white dot represents the median, the black bar depicts the interquartile range (IQR) and whiskers extend 1.5 times the IQR past the quartiles.

**Supplementary Figure S4A:** Distribution of overall pneumococcal carriage density (log_10_ GE/mL) by A) age in months (n=321), B) number of serotypes (n=279), C) prior antibiotic use (n=319), D) household crowding (n=311), E) pneumonia severity (n=305), F) type of cooking fuel used (n=316), G) smoker in house (n=316), H) season (n=321), and I) malnutrition status (n=316) among unvaccinated children aged 2-59 months with pneumococcal carriage admitted with acute respiratory infections in Mongolia.

The white dot represents the median, the black bar depicts the interquartile range (IQR) and whiskers extend 1.5 times the IQR past the quartiles.

**Supplementary Figure S4B:** Distribution of PCV13-type pneumococcal carriage density (log_10_ GE/mL) by A) age in months (n=170), B) number of serotypes (n=170), C) prior antibiotic use (n=168), D) household crowding (n=166), E) pneumonia severity (n=161), F) type of cooking fuel used (n=168), G) smoker in house (n=168), H) season (n=170), and I) malnutrition status (n=166) among unvaccinated children aged 2-59 months with pneumococcal carriage admitted with acute respiratory infections in Mongolia.

The white dot represents the median, the black bar depicts the interquartile range (IQR) and whiskers extend 1.5 times the IQR past the quartiles.

**Supplementary Figure S4C:** Distribution of non-PCV13 type pneumococcal carriage density (log_10_ GE/mL) by A) age in months (n=23), B) number of serotypes (n=23), C) prior antibiotic use (n=23), D) household crowding (n=23), E) pneumonia severity (n=23), F) type of cooking fuel used (n=23), G) smoker in house (n=23), H) season (n=23), and I) malnutrition status (n=23) among unvaccinated children aged 2-59 months with pneumococcal carriage admitted with acute respiratory infections in Mongolia. The white dot represents the median, the black bar depicts the interquartile range (IQR) and whiskers extend 1.5 times the IQR past the quartiles.

**Supplementary Figure S5A. Serotype-specific density distribution of some of the common serotypes by PCV13 vaccination and number of serotypes in children with pneumonia and pneumococcal carriage in Papua New Guinea.** Blue= Single serotype; Pink= Multiple serotypes. A-E= PCV13 VTs; F-J=non-PCV13 VTs. Boxes depict the interquartile range (IQR) with a central line at the median, and whiskers extend 1.5 times the IQR past the quartiles. Values outside whiskers plotted as individual points.

**Supplementary Figure S5B. Serotype-specific density distribution of some of the common serotypes by PCV13 vaccination and number of serotypes in children with acute respiratory infection and pneumococcal carriage in Lao People’s Democratic Republic.** Blue= Single serotype; Pink= Multiple serotypes. A-E= PCV13 VTs; F-J= non-PCV13 VTs. Boxes depict the interquartile range (IQR) with a central line at the median, and whiskers extend 1.5 times the IQR past the quartiles. Values outside whiskers plotted as individual points.

**

**Supplementary Figure S5C. Serotype-specific density distribution of some of the common serotypes by PCV13 vaccination and number of serotypes in children with pneumonia and pneumococcal carriage in Mongolia.** Blue= Single serotype; Pink= Multiple serotypes. A-F= PCV13 VTs; G-J= non-PCV13 VTs. NT2 and NT3b refer to different lineages of non-encapsulated pneumococci. Boxes depict the interquartile range (IQR) with a central line at the median, and whiskers extend 1.5 times the IQR past the quartiles. Values outside whiskers plotted as individual points.

**References**

1. Chan J, Nguyen CD, Lai JYR, et al. Determining the pneumococcal conjugate vaccine coverage required for indirect protection against vaccine-type pneumococcal carriage in low and middle-income countries: a protocol for a prospective observational study. BMJ Open 2018; 8(5): e021512.

2. von Mollendorf C, Mungun T, Ulziibayar M, et al. Effect of pneumococcal conjugate vaccination on pneumococcal carriage in hospitalised children aged 2–59 months in Mongolia: an active pneumonia surveillance programme. The Lancet Microbe 2024: 100929.

3. Satzke C, Dunne EM, Choummanivong M, et al. Pneumococcal carriage in vaccine-eligible children and unvaccinated infants in Lao PDR two years following the introduction of the 13-valent pneumococcal conjugate vaccine. Vaccine 2019; 37(2): 296-305.

4. Britton KJ, Pickering JL, Pomat WS, et al. Lack of effectiveness of 13-valent pneumococcal conjugate vaccination against pneumococcal carriage density in Papua New Guinean infants. Vaccine 2021; 39(38): 5401-9.

5. Satzke C, Turner P, Virolainen-Julkunen A, et al. Standard method for detecting upper respiratory carriage of *Streptococcus pneumoniae*: updated recommendations from the World Health Organization Pneumococcal Carriage Working Group. Vaccine 2013; 32(1): 165-79.

6. Satzke C, Dunne EM, Porter BD, et al. The PneuCarriage Project: A Multi-Centre Comparative Study to Identify the Best Serotyping Methods for Examining Pneumococcal Carriage in Vaccine Evaluation Studies. PLoS Med 2015; 12(11): e1001903.

7. Salter SJ, Hinds J, Gould KA, et al. Variation at the capsule locus, cps, of mistyped and non-typable *Streptococcus pneumoniae* isolates. Microbiology 2012; 158(6): 1560-9.

8. Manna S, Ortika BD, Dunne EM, et al. A novel genetic variant of Streptococcus pneumoniae serotype 11A discovered in Fiji. Clin Microbiol Infect 2018; 24(4): 428 e1- e7.
